# Effectiveness of a quality improvement collaborative in reducing time to surgery for patients requiring emergency cholecystectomy

**DOI:** 10.1002/bjs5.50221

**Published:** 2019-10-08

**Authors:** J. R. Bamber, T. J. Stephens, D. A. Cromwell, E. Duncan, G. P. Martin, N. F. Quiney, J. F. Abercrombie, I. J. Beckingham, on behalf of the Cholecystectomy Quality Improvement Collaborative, J. Abraham, I. Ahmad, J. Ahmed, M. Andrews, B. Appleton, M. Asif, R. Bolton, C. Briggs, U. Bumagat, S. Burchfield, G. Cochrane, F. Dewi, G. Dovell, S. Dyer, J. Edge, R. Edwards, I. Fabre, E. Gemmill, E. Griffiths, D. Hariharan, E. Harrington‐Patel, A. Hassn, M. Hepworth, J. Hewes, S. Hine, M. Hollyman, K. Ide, D. Jenner, R. Johnson, S. Jordan, S. Karamanakos, J. Kovoor, N. Kukreja, G. Marangoni, N. Metcalfe, P. Morcous, P. Needham, N. Patel, N. Qureshi, N. Rajaretnam, I. Rajendran, Y. Sabah, D. L. Sanders, A. Sandison, J. Sansom, R. Seth, V. Shetty, T. Sollei, S. Strong, L. Sullivan, R. P. Sutcliffe, L. Talbot, G. Taylor, V. Varadarajan, E. Villatoro, J. Wardale, S. Weaver, T. Wiggins, A. Wood

**Affiliations:** ^1^ Practicality Consulting Queen Mary University of London London UK; ^2^ William Harvey Research Institute Queen Mary University of London London UK; ^3^ Department of Health Services Research and Policy London School of Hygiene and Tropical Medicine London UK; ^4^ Department of Professional Standards Royal College of Surgeons of England London UK; ^5^ The Healthcare Improvement Studies (THIS) Institute University of Cambridge Cambridge UK; ^6^ Department of Anaesthesia Royal Surrey County Hospital Guildford UK; ^7^ Departments of Colorectal Surgery Queen's Medical Centre Nottingham UK; ^8^ Hepatobiliary and Pancreatic Surgery Queen's Medical Centre Nottingham UK

## Abstract

**Background:**

Acute gallstone disease is a high‐volume emergency general surgery presentation with wide variations in the quality of care provided across the UK. This controlled cohort evaluation assessed whether participation in a quality improvement collaborative approach reduced time to surgery for patients with acute gallstone disease to fewer than 8 days from presentation, in line with national guidance.

**Methods:**

Patients admitted to hospital with acute biliary conditions in England and Wales between 1 April 2014 and 31 December 2017 were identified from Hospital Episode Statistics data. Time series of quarterly activity were produced for the Cholecystectomy Quality Improvement Collaborative (Chole‐QuIC) and all other acute National Health Service hospitals (control group). A negative binomial regression model was used to compare the proportion of patients having surgery within 8 days in the baseline and intervention periods.

**Results:**

Of 13 sites invited to join Chole‐QuIC, 12 participated throughout the collaborative, which ran from October 2016 to January 2018. Of 7944 admissions, 1160 patients had a cholecystectomy within 8 days of admission, a significant improvement (*P* < 0·050) from baseline performance. This represented a relative change of 1·56 (95 per cent c.i. 1·38 to 1·75), compared with 1·08 for the control group. At the individual site level, eight of the 12 Chole‐QuIC sites showed a significant improvement (*P* < 0·050), with four sites increasing their 8‐day surgery rate to over 20 per cent of all emergency admissions, well above the mean of 15·3 per cent for control hospitals.

**Conclusion:**

A surgeon‐led quality improvement collaborative approach improved care for patients requiring emergency cholecystectomy.

## Graphical abstract

Evaluation was undertaken to determine whether a 12‐hospital quality improvement collaborative could be effective in assisting surgeons to implement national guidance to improve the care pathway for patients with acute gallstone disease. A negative binomial regression model showed a significant improvement in the 8‐day surgery rate for participating hospitals (in line with national guidance), above any national trend of all English and Welsh hospitals. A surgeon‐led quality improvement collaborative approach is effective at improving care for patients requiring emergency cholecystectomy.

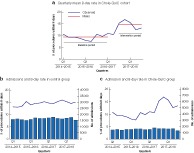

Evidence of the value of a quality improvement programme

## Abstract (es)

**Antecedentes:**

La patología biliar aguda litiásica es una de las urgencias con más volumen de casos en cirugía general, con amplias variaciones en la calidad de la atención prestada en todo el Reino Unido. En este estudio de cohortes controlado se valoró si la participación en un enfoque colaborativo de mejora de la calidad disminuía el tiempo hasta la cirugía en pacientes con patología biliar aguda litiásica a menos de 8 días desde la presentación, de acuerdo con la guía nacional.

**Métodos:**

Se identificó a los pacientes que precisaron un ingreso hospitalario por patología biliar aguda en Inglaterra y Gales, del 1 de abril de 2014 al 31 de diciembre de 2017, a partir de datos de las estadísticas de episodios hospitalarios. Se crearon series temporales de actividad trimestral para Chole‐QuIC y para todos los demás hospitales de agudos del NHS (grupo control). Se utilizó un modelo de regresión binomial negativa para comparar la proporción de pacientes sometidos a cirugía dentro de los primeros 8 días en los periodos basal y de intervención.

**Resultados:**

De los 13 sitios invitados a unirse a Chole‐QuIC, 12 participaron durante toda la colaboración, que se desarrolló entre octubre de 2016 y enero de 2018. De los 7.944 ingresos, en 1.160 pacientes se realizó la colecistectomía dentro de los 8 días posteriores a su ingreso, una mejora significativa (*P* < 0,05) en comparación con el periodo previo a la intervención. Esto representó un cambio relativo de 1,56 (i.c. del 95%: 1,38 a 1,75) en comparación con 1,08 para el grupo de control. A nivel de cada uno de los hospitales, ocho de los 12 centros Chole‐QuIC presentaron una mejora significativa (*P* < 0,05), y en cuatro de ellos el porcentaje de cirugía en 8 días aumentó a más del 20% de todos los ingresos urgentes, muy por encima del promedio de 15,3% para hospitales de control.

**Conclusión:**

Un enfoque colaborativo de mejora de la calidad dirigido por el cirujano mejoró la atención a los pacientes que precisan una colecistectomía urgente.

## Introduction

Gallstone‐related disease accounts for approximately one‐third of emergency general surgery admissions and referrals^1^. The commonest presentation is acute biliary pain (56 per cent), followed by acute cholecystitis (36 per cent) and gallstone pancreatitis (4 per cent). The majority of patients presenting to hospital with biliary pain go on to have a cholecystectomy as definitive treatment. Around 20–33 per cent of patients with acute cholecystitis or pancreatitis will re‐present with gallstone‐related symptoms before they have a cholecystectomy^2^, ^3^, ^4^. Current national guidance from the UK National Institute for Health and Care Excellence (NICE) is for laparoscopic cholecystectomy to be done within 7 days of a diagnosis of acute cholecystitis, and within the index admission for pancreatitis^5^. Guidelines from the International Hepato‐Pancreato Biliary Association, World Society of Emergency Surgery and British Society of Gastroenterologists provide similar guidance on times to cholecystectomy^6^, ^7^, ^8^.

Reducing the time to surgery for people who need a cholecystectomy minimizes the number of times patients are readmitted with the same diagnosis and decreases the overall length of hospital stay. Compared with delayed cholecystectomy, emergency procedures are associated with overall fewer work‐days lost, greater patient satisfaction and better quality of life^3^. Patients with acute pancreatitis run the risk of a fatal episode whilst awaiting cholecystectomy that can be reduced by early biliary surgery. Concerns of complications resulting from conversion from laparoscopic to open surgery or increased risks of bile duct injury with emergency compared with delayed surgery are not supported by current evidence^9^, ^10^, ^11^, ^12^, ^13^, ^14^, ^15^, ^16^, ^17^, ^18^, ^19^. Several studies have tried to identify the optimal time within the first week of admission, and some^14^, ^15^, though not all^20^, have shown a lower conversion rate if surgery is possible within 72 h. Meta‐analysis of available studies has not shown a higher incidence of bile duct injury, which remains similar to that in patients having delayed surgery^21^, ^22^, ^23^. Across studies^20^, ^21^, ^22^, ^23^, ^24^, patients who have surgery after 72 h still do better on all indicators than those in delayed groups.

Patients in the UK with symptomatic gallstones wait longer and are more likely to be readmitted than those in many other countries. Patients in France, the USA and Australia tend to undergo cholecystectomy on first admission with an average length of stay under 36 h^15^, ^25^, ^26^. The majority of patients diagnosed with acute cholecystitis in the USA have an emergency cholecystectomy on the same admission, compared with only 16 per cent in England^1^. Within the UK there is wide variation between National Health Service (NHS) hospitals in the management of these patients^27^, and wide variation in cholecystectomy rates despite NICE guidance^5^.

The goal of the Cholecystectomy Quality Improvement Collaborative (Chole‐QuIC) was to see whether a collaborative quality improvement (QI) programme could be used to improve emergency cholecystectomy rates. The evaluation was designed to identify whether improvements could be achieved by hospitals as a result of participation in Chole‐QuIC, what influenced change, and what lessons might be drawn for future improvement efforts. This article presents the quantitative outcome evaluation findings, using routinely collected administrative hospital data to answer the question: ‘Did participation in a quality improvement collaborative (Chole‐QuIC) reduce time to surgery to within 8 days from admission for patients requiring emergency cholecystectomy?’. The findings from the process evaluation have been published separately^28^.

## Methods

The Chole‐QuIC study ran from October 2016 to January 2018 as a modified version of the Institute of Healthcare Improvement Breakthrough Series collaborative approach, incorporating evidence related to this QI approach (*Table* 1)^29^, ^30^, ^31^, ^32^. Recruitment to the collaborative was through open application, followed by a selection process. Some 13 of the 29 hospitals that applied were selected to participate. Criteria for selection involved: the ability of the site to commit sufficient surgical and support time to the programme; and sufficient room for improvement and no ongoing related improvement projects. The selected hospitals covered a range of hospital size and surgical volume, with three sites that also provided specialist hepatobiliary services.

**Table 1:** The Chole‐QuIC process for developing and delivering an evidence‐based quality improvement collaborative

**The right problem**
Choosing a problem with common agreement that needs fixing in this context (defined by stakeholders) and motivation from participants to solve
Clearly defining and articulating the problem
**Measuring and monitoring**
Data collection, to understand the local demand, the size of the challenge and patient flow through the actual pathway
Data analysis and feedback to monitor progress and motivate colleagues
**Support and collaboration**
Sharing of ideas and outcomes with the collaborative; learning from other attempts and adapting local processes accordingly
Expert clinical and quality improvement support, training and coaching
**The right solutions**
Generating context‐specific solutions or new processes (supported by best evidence of any previous solutions)
Testing these solutions, and adapting to what works well or does not

The aim of the collaborative was to demonstrate that time to emergency cholecystectomy could be reduced for eligible patients with acute biliary pain, cholecystitis or gallstone pancreatitis, by using QI methodologies to enable clinicians to drive change within their own institutions. Surgery within 8 days of presentation was chosen to match current NICE guidelines for acute cholecystitis (surgery within 7 days of diagnosis), plus one additional day from presentation to allow time for diagnosis^5^. Eligible patients were those who agreed to have their operation ( cholecystectomy) on an emergency basis and were deemed to be clinically fit for surgery, as assessed by local clinical teams. This patient population was chosen as it covered the majority of patients with symptomatic gallstones following a similar clinical pathway^1^.

**Figure 1:**
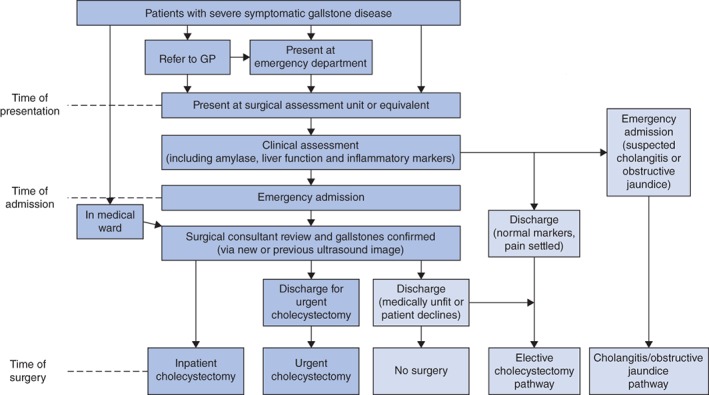
Chole‐QuIC pathway for patients with acute biliary pain, cholecystitis or gallstone pancreatitis Lighter coloured boxes on the right relate to patients who drop out of the Chole‐QuIC pathway because further diagnostic information is received or the patient chooses not to have an emergency cholecystectomy. GP, general practitioner.

*Fig*. 1 shows the clinical pathway for patients requiring emergency cholecystectomy. There was no evidence to suggest that a single, universally applicable, organizational approach would achieve this goal; rather, successful change would require concerted short‐term resources to implement behavioural, process and system improvements. Consequently, the collaborative was designed to support site teams to develop, test and ultimately implement context‐specific solutions that would move their service toward achieving the project goal. Teams were supported throughout the collaborative process in a number of ways (*Table* 1). A series of four collaborative meetings, designed to help teams learn about improvement methods and share progress and ideas with one another, was organized; webinars between meetings were used to maintain momentum and to keep each centre updated; and site visits were undertaken by the Chole‐QuIC team to support teams to make improvements and overcome specific challenges. The Chole‐QuIC team also provided assistance with analysis of locally collected audit data, to help teams track their own progress. Full details of the Chole‐QuIC intervention have been published elsewhere^28^.

### Study design

A mixed‐methods evaluation was approved by the ethics review board of Queen Mary University of London (QMREC1817a). NHS Research Ethics Committee approval was not required for the analysis of anonymized routine data for service evaluation. Project findings are reported in accordance with Standards for QUality Improvement Reporting Excellence (SQUIRE) guidelines^33^ for the publication of QI work.

Informed consent was obtained from all participants and written information was provided at the start of the programme about the purpose of the evaluation, the voluntary nature of participation, and assurances that no individual or hospital‐level data would be disclosed.

### Data collection

This evaluation used routine hospital data on all patients admitted as an emergency with acute biliary pain, cholecystitis or gallstone pancreatitis (ICD‐10 codes K85.0, K85.1, K85.8, K85.9; K80.0, K80.1, K80.2; K81.0, K81.1, K81.8, K81.9; K82.0, K82.1, K82.2, K82.3, K82.4, K82.8, K82.9; R10) who subsequently had a cholecystectomy, from 1 April 2014 to 31 December 2017. For English NHS trusts, aggregate quarterly figures were derived from the English Hospital Episode Statistics database; for Welsh Health Boards, data were obtained from the Patient Episode Database for Wales. Key variables were the number of patients admitted as an emergency with an eligible condition and the number of patients who had emergency or elective surgery (OPCS J18) within 8 days of emergency admission. Some quarterly values were masked as ‘< 5’; for analysis, values between 1 and 4 for the masked values were imputed by multiplying the emergency admissions with the typical ratio for that NHS trust (up to a maximum of 4 patients).

QI data were collected by hospital teams for all patients on the Chole‐QuIC clinical pathway (*Fig*. 1), including patient eligibility, time to surgery, and whether the patient had an inpatient cholecystectomy, was discharged for an urgent cholecystectomy, or was temporarily or permanently unfit for surgery. Anonymized summary data were shared with the Royal College of Surgeons core team, using an encrypted e‐mail service. Run charts and statistical process control charts were created from these summary data to assess local improvements, including changes to mean and median times to surgery, and 3‐ and 14‐day surgery rates. These data were fed back to teams monthly, starting 3 months before the start of the study, to support improvement, and were analysed as part of the evaluation.

### Data analysis

Time series of quarterly activity were produced for: each of the English and Welsh NHS organizations participating in Chole‐QuIC; the English and Welsh site Chole‐QuIC cohort as a whole; and a combined English and Welsh control group, consisting of 127 English acute NHS trusts and five Welsh Health Boards.

The time series was divided into three segments: nine quarters from April 2014 to June 2016 represented the baseline period (baseline); one quarter from July to September 2016 was considered a transition period; and five quarters from October 2016 to December 2017 represented the intervention period (intervention). A negative binomial regression model was used to assess whether the proportion of patients having surgery within 8 days had changed in the intervention compared with the baseline period. The model only assessed whether there had been a change in the mean level of the time series, because it was too short to test for (changes in) trends. A second model, with the relative difference adjusted for the change observed in the control group, was also used. A negative binomial regression model was preferred to a Poisson model as it allowed for overdispersion.

A statistical process control (SPC) chart was created from the time to surgery for all eligible patient admissions that had a cholecystectomy at any of the 12 participating sites, using locally collected data. The analysis was undertaken using Stata® version 15.1 (StataCorp, College Station, Texas, USA). Time to surgery was plotted by date of presentation, and upper and lower control limits were calculated at three standard deviations from the mean time to surgery. Following standard practice for SPC chart interpretation, mean and control limits were recalculated when a shift (9 or more successive data points above or below the mean) was identified. A shift is a data signal in an SPC chart that indicates a special cause variation, analogous to a significant, non‐random, change in the data (*P* < 0·050)^34^, ^35^.

## Results

Of the 13 sites invited to join Chole‐QuIC, 12 participated fully throughout the programme, with teams attending all four collaborative meetings, three webinars, participating in at least one site visit, collecting prospective data and testing improvement ideas. Site 13 withdrew voluntarily after 9 months, having engaged to only a limited extent (no attempt at service changes, incomplete local data collection), and was included only in the control group in the main analyses.

In total there were just under 8000 acute biliary admissions across the Chole‐QuIC cohort of 12 during the 15‐month intervention period: 5390 with acute biliary pain or cholecystitis, and 2554 with acute pancreatitis. Some 1160 patients had a cholecystectomy within 8 days of admission, 428 more than in the previous 15 months.

**Figure 2:**
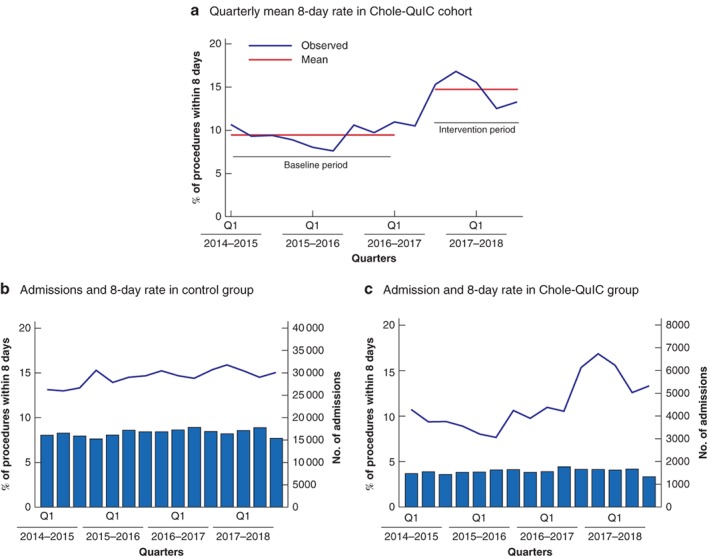
Admissions and 8‐day rates for baseline and intervention periods in Chole‐QuIC cohort and control group **a** Time series of quarterly mean 8‐day rate in the Chole‐QuIC cohort. **b,c** Comparison of emergency biliary admissions by quarter and 8‐day cholecystectomy rate in **b** control and **c** intervention (Chole‐QuIC) sites.

A significant increase in the 8‐day surgery rate was identified, above any national trend toward improvement, for sites participating in Chole‐QuIC, with an increase in the quarterly mean of emergency cholecystectomies for all 12 sites, from 145·5 in the baseline period to 232·0 in the intervention period. Although the number of emergency admissions stayed relatively stable for both the Chole‐QuIC and the control group (the other 127 hospitals across England and 5 Health Boards Wales), the percentage of procedures within 8 days rose, with the control group 8‐day rate increasing slightly (from 14·2 to 15·3 per cent) and the Chole‐QuIC group increasing markedly, from a mean of 9·4 per cent during the baseline period to 14·6 per cent in the intervention period (*Fig*. 2). This improvement represented a relative change of 1·56, compared with 1·08 for the control group; accounting for the national trend towards improvement in the control group gives a relative change of 1·45 (95 per cent c.i. 1·29 to 1·62) (*Table* 2).

**Table 2:** Chole‐QuIC and control 8‐day surgery rates and individual site data

	Activity (all admissions for biliary disease)		% of procedures within 8 days (all admissions)			
	Baseline	Intervention	Baseline	Intervention	Relative change from baseline	Relative change for combined model (adjusted for control group)
**All Chole‐QuIC**	13 929	7944	9·4	14·6	1·56 (1·38, 1·75)*	1·45 (1·29, 1·62)*
**Control**	147 495	83 391	14·2	15·3	1·08 (1·02, 1·14)*	
**Site no.**						
1	521	301	8·8	25·9	2·94 (2·02, 4·27)*	2·73 (1·88, 3·96)*
2	964	521	12·2	26·5	2·16 (1·69, 2·77)*	2·01 (1·55, 2·60)
3	513	355	16·8	35·2	2·10 (1·60, 2·76)*	1·95 (1·47, 2·59)
4	1103	629	9·9	20·8	2·09 (1·45, 3·01)*	1·96 (1·50, 2·55)*
5	1333	770	4·6	8·6	1·88 (1·27, 2·77)*	1·74 (1·22, 2·49)*
6	1114	619	8·5	14·7	1·72 (1·06, 2·79)*	1·60 (1·19, 2·16)*
7	1189	627	6·7	11·2	1·68 (1·06, 2·65)*	1·54 (1·11, 2·15)*
8	1413	900	14·4	19·6	1·35 (1·11, 1·66)*	1·26 (1·01, 1·56)*
9	1213	684	6·5	8·3	1·28 (0·88, 1·85)	1·19 (0·84, 1·68)
10	1476	760	8·4	8·8	1·03 (0·64, 1·66)	0·97 (0·72, 1·33)
11	1505	793	2·9	3·0	1·02 (0·59, 1·77)	0·96 (0·58, 1·59)
12	1585	985	16·5	14·2	0·86 (0·69, 1·09)	0·80 (0·64, 100)

Values in parentheses are 95 per cent confidence intervals.

* *P* < 0·050 (negative binomial regression).

**Figure 3:**
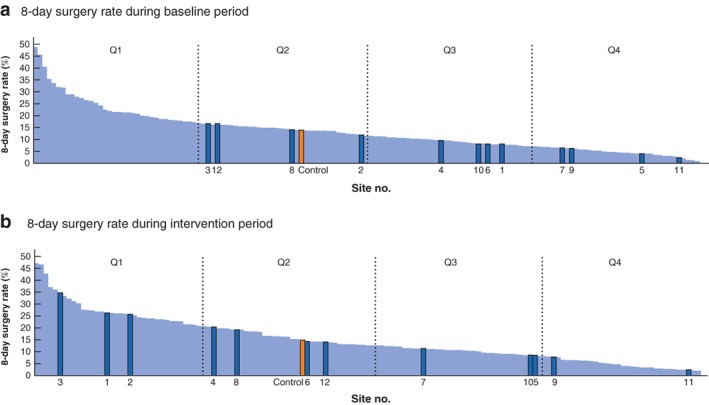
Percentage of procedures within 8 days of admission in baseline and intervention periods Performance (8‐day surgery rate) during **a** baseline period and **b** intervention period for the 12 participating sites. The control group mean performance represents data from Chole‐QuIC sites in the national context. Q, quarter.

At the individual site level, eight of the 12 sites had a significant improvement (*P* < 0·050) in the 8‐day surgery rate above the national trend towards improvement; in four sites, the 8‐day surgery rate increased to over 20 per cent of all emergency admissions (*Table* 2). As a comparator, between 2014 and 2018, 8‐day surgery rates were between 2 and 48 per cent across England and Wales, with the top quartile across England and Wales achieving a median of 26 (range 21–48) per cent. *Fig*. 3 illustrates the ranking of Chole‐QuIC sites for the 8‐day surgery rate compared with all English and Welsh trusts over the baseline and intervention periods. All but two (sites 11 and 12) of the Chole‐QuIC sites improved their ranking in emergency surgery rates among English and Welsh hospitals (*Fig*. 3), with three moving into the top quartile and two of three moving from the fourth to third quartiles. Sites 11 and 12 saw concurrent reductions in performance.

**Figure 4:**
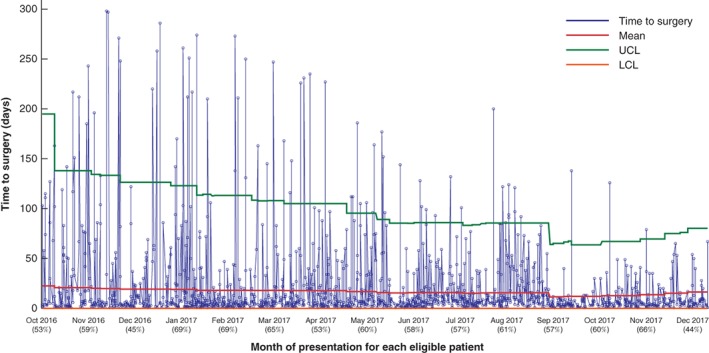
Statistical process control chart for all patients who had surgery in the 12 participating sites Data on time to surgery for the 1580 fit and eligible patients who had a cholecystectomy after emergency admission during the improvement period (from the day after the launch, 7 October 2016, to 30 December 2017). Values in parentheses below each month indicate the percentage of eligible patients who had surgery. UCL, upper control limit; LCL, lower control limit.

*Fig*. 4 presents the locally collected data on time to surgery for the 1580 patients who had a cholecystectomy following emergency admission, from a total of 3001, during the improvement period. Although the percentage of eligible patients undergoing cholecystectomy remained consistent throughout the project (ranging from 44 to 69 per cent), the mean time to surgery for these patients improved over time, and variation reduced. Mean time to surgery decreased from 22·6 days at the project start to a low of 12·0 days (September to October 2017), finishing at 16·5 days in December 2017. Variation in time to surgery also reduced substantially over time: from September 2017, breaches to the upper control limit became rare, despite a concurrent tightening of the control limits.

## Discussion

The main finding of this evaluation was that eight of 12 hospitals participating in a QI collaborative were able to increase early cholecystectomy rates significantly for patients with gallstone disease requiring hospital admission, in line with national guidance. As this was a controlled cohort evaluation, it was possible to account for any trend towards improvement in the remaining 127 hospitals in England and Wales. Results remained significant when the small national improvement trend was accounted for. When plotted on SPC charts, locally collected data showed a clear reduction in variability and increase in reliability in providing timely laparoscopic cholecystectomy across the Chole‐QuIC cohort.

Regarding the 8‐day surgery rate, the cohort mean of 14·6 per cent remained below the national control group average of 15·3 per cent. This is explained partly by the large range of outcomes between the 12 (3·0–35·2 per cent) and partly by the selection criteria for inclusion in the programme. Sites were chosen with room for improvement that were not currently involved in improvement activities in this area. It is important to stress that this was an improvement programme where changes were introduced throughout the 15 months, not introduced in totality at the start. When interpreting these rates, only five of the 127 English trusts and no Welsh hospitals achieved an 8‐day surgery rate above 35 per cent between 2014 and 2017 (*Fig*. 3). These data may appear low as the denominator includes all patients recorded with relevant ICD‐10 codes. Clinical assessments by the participating site teams suggested that a large proportion of patients in this denominator group were not eligible for surgery. For patients who were suitable for early surgery, this study showed that it was possible for hospitals to improve their service significantly to ensure these patients received timely surgery.

The variation in outcomes between the 12 sites suggests that there are particular factors that aid or hinder successful improvement. Several important themes emerged from the concurrent mixed‐methods process evaluation, which looked at the characteristics of the four most successful sites compared with the four least successful sites (*Table* 3)^28^.

**Table 3:** Description of key influences

**Achieving clarity of purpose amongst site leads and all key stakeholders**
**Creating additional capacity to do urgent cholecystectomies. Solutions include:**
Ring‐fencing half‐day elective lists for hot gallbladders
Persuading additional surgeons to carry out operations on hot gallbladders
Holding a slot on emergency theatre lists (CEPOD) for hot gallbladders
**Coordinating/managing the patient pathway effectively. Solutions include:**
E‐mail referral system
Real‐time review systems, such as whiteboard in surgical assessment unit listing details of all eligible admissions
Virtual wards
**Other cognitive, relational and behavioural work**
Capacity (time and resources) to lead and effective team working
Ideas to action – e.g. testing ideas quickly
Learning from own and others' experience – e.g. changing approaches upon review, adding new innovations over time

CEPOD, Confidential Enquiry into Perioperative Deaths.

Intensive work was required to ensure that all stakeholders within teams had a shared understanding of, and agreement with, the purpose and benefits of rapid surgical intervention for this patient group. Divergent views on the value of this approach amongst key stakeholders (surgeons, senior service managers and staff gatekeeping emergency theatre lists) seemed to be the most significant barrier to improvement. At many sites, these patients were seen as comparable to patients presenting with acute appendicitis, and the Chole‐QuIC sites that improved most focused on changing the local culture and approach in relation to this patient group.

Sites exhibiting the greatest improvement used a multifaceted approach to creating additional theatre capacity, for example improving use of emergency lists while also ring‐fencing slots within elective lists to provide urgent surgery for these emergency admissions. An effective coordination process for patients moving along the commonly agreed pathway was important to optimize the use of any additional capacity created and to allay concerns about potential waste from ring‐fencing.

Cognitive, relational and behavioural issues, such as having dedicated time for site leads to run the improvement project locally and turning ideas into action early on in the project, were found to define the most successful sites. These four interlinked factors needed to be developed over time. Achieving clarity of purpose appeared a necessary precondition. The more challenged sites failed to achieve sufficient stakeholder support from all parties, which hampered their ability to implement improvements to capacity and coordination processes^28^.

National gallstone registries in both Sweden and Denmark have been credited with facilitating overall improvements in quality of care through benchmarking^36^, ^37^, but more active efforts to use QI methods have not been undertaken. Surgeon‐led improvement collaboratives appear rare, with a recent systematic review^38^ of published improvement collaborative evaluations finding that only a small number focused on improving care for surgical patients, and only one in general surgery. With regard to the success of this collaborative, the robustness of the evaluation design and the results achieved compare favourably with the outcomes of previous QI collaboratives in other areas of healthcare. Although this is likely to be due, in part, to an effective evidence‐based intervention design, the motivation and efforts of the site teams are likely to be a major contributor to the relative success of Chole‐QuIC. This reinforces the argument that QI collaboratives should focus on an issue or problem for which there is a strong consensus that change is required, especially when both problem and solution identification are supported by a respected professional body^28^, ^32^.

This evaluation has several strengths, including the use of registry data that facilitated both a substantial baseline data period and comparison with a control group to observe both cohort and national trends toward improvements in care for this patient group. The evaluation also has limitations. Sites volunteered to participate and thus a commitment to provide surgical and support time for the duration of the project was present in the sites that may not be generalizable. To demonstrate that improvements could be achieved and sustained in a range of contexts, the programme was designed to select sites with capacity for improvement. Most sites therefore had baseline performance with regard to the 8‐day rate below the national average. As five of the eight members of the evaluation team were directly involved in delivering this programme, there is a risk of bias in the analysis, which was mitigated by ensuring that an independent researcher carried out the quantitative analysis and an independent evaluation expert regularly reviewed processes to provide both internal and face validity. Some potentially relevant outcome data were not available for analysis from routine national data, including readmission rates, 14‐day rates, or median and mean time to surgery. Balancing measures such as positive or negative impact on waiting times for other patient groups were not looked at, and neither was the sustainability of these outcomes, as national data were available only to December 2017.

A surgeon‐led QI collaborative approach can be effective at improving care for patients requiring emergency cholecystectomy. The learning from this collaborative should be useful for others wishing to improve care for patients with acute gallstone‐related disease and potentially for other surgical patient groups where current care is below the standards set by national guidance.

## Collaborators

Other members of the Cholecystectomy Quality Improvement Collaborative (Chole‐QuIC): J. Abraham, I. Ahmad, J. Ahmed, M. Andrews, B. Appleton, M. Asif, R. Bolton, C. Briggs, U. Bumagat, S. Burchfield, G. Cochrane, F. Dewi, G. Dovell, S. Dyer, J. Edge, R. Edwards, I. Fabre, E. Gemmill, E. Griffiths, D. Hariharan, E. Harrington‐Patel, A. Hassn, M. Hepworth, J. Hewes, S. Hine, M. Hollyman, K. Ide, D. Jenner, R. Johnson, S. Jordan, S. Karamanakos, J. Kovoor, N. Kukreja, G. Marangoni, N. Metcalfe, P. Morcous, P. Needham, N. Patel, N. Qureshi, N. Rajaretnam, I. Rajendran, Y. Sabah, D. L. Sanders, A. Sandison, J. Sansom, R. Seth, V. Shetty, T. Sollei, S. Strong, L. Sullivan, R. P. Sutcliffe, L. Talbot, G. Taylor, V. Varadarajan, E. Villatoro, J. Wardale, S. Weaver, T. Wiggins, A. Wood.
